# Molecular Cloning and Characterization of *DXS* and *DXR* Genes in the Terpenoid Biosynthetic Pathway of *Tripterygium wilfordii*

**DOI:** 10.3390/ijms161025516

**Published:** 2015-10-23

**Authors:** Yuru Tong, Ping Su, Yujun Zhao, Meng Zhang, Xiujuan Wang, Yujia Liu, Xianan Zhang, Wei Gao, Luqi Huang

**Affiliations:** 1 School of Traditional Chinese Medicine, Capital Medical University, Beijing 100069, China; E-Mails: tongyuru13@126.com (Y.T.); suping120@163.com (P.S.); diana1989119@sina.com (Y.Z.); zhangmeng8846@163.com (M.Z.); tmwangxj@ccmu.edu.cn (X.W.); lyjlife@sina.com (Y.L.); xnzhang@ccmu.edu.cn (X.Z.); 2 National Resource Center for Chinese Materia Medica, China Academy of Chinese Medical Sciences, Beijing 100700, China

**Keywords:** *Tripterygium wilfordii*, *TwDXS1*, *TwDXS2*, *TwDXR*, color complementation

## Abstract

1-Deoxy-d-xylulose-5-phosphate synthase (*DXS*) and 1-deoxy-d-xylulose-5-phosphate reductoisomerase (*DXR*) genes are the key enzyme genes of terpenoid biosynthesis but still unknown in *Tripterygium wilfordii* Hook. f. Here, three full-length cDNA encoding DXS1, DXS2 and DXR were cloned from suspension cells of *T. wilfordii* with ORF sizes of 2154 bp (*TwDXS1*, GenBank accession no.KM879187), 2148 bp (*TwDXS2*, GenBank accession no.KM879186), 1410 bp (*TwDXR*, GenBank accession no.KM879185). And, the *TwDXS1*, *TwDXS2* and *TwDXR* were characterized by color complementation in lycopene accumulating strains of *Escherichia coli*, which indicated that they encoded functional proteins and promoted lycopene pathway flux. *TwDXS1* and *TwDXS2* are constitutively expressed in the roots, stems and leaves and the expression level showed an order of roots > stems > leaves. After the suspension cells were induced by methyl jasmonate, the mRNA expression level of *TwDXS1*, *TwDXS2*, and *TwDXR* increased, and triptophenolide was rapidly accumulated to 149.52 µg·g^−1^, a 5.88-fold increase compared with the control. So the *TwDXS1*, *TwDXS2*, and *TwDXR* could be important genes involved in terpenoid biosynthesis in *Tripterygium wilfordii* Hook. f.

## 1. Introduction

*Tripterygium wilfordii* (Hook. f.) is a woody vine of the Celastraceae family, and native to China (south of the Yangtze River), Korea, and Japan [1]. As a kind of Chinese medicinal herb, called Lei gong teng, it has been used for treatment of chills, edema and carbuncle, and has a variety of pharmaceutical activities, such as anti-rheumatoid arthritis [2,3], mediating cancer cell death [4,5], and anti-HIV activity [6]. Terpenoids are the main bioactive components in *T. wilfordii*, such as triptophenolide, triptolide, and celastrol [7,8,9]. The triptophenolide shows obvious inhibiting effects on lymphocyte and thymus dependent antibody IgG [10], and can be used as starting material to synthesize triptolide and triptonide [11].

The biosynthesis of terpenoids includes the common precursor substance isopentenyl diphosphate (IPP) and its isomer dimethylallyl diphosphate (DMAPP), which are generated through two pathways: the mevalonate (MVA) pathway and the methylerythritol phosphate (MEP) pathway [12]. The MEP pathway is principally involved in providing isoprenoids for monoterpenes [13], diterpenes [14] and carotenoids [15]. The 1-deoxy-d-xylulose-5-phosphate synthase (*DXS*) and 1-deoxy-d-xylulose-5-phosphate reductoisomerase (*DXR*) genes are the first two rate-limiting enzyme genes in this pathway. DXS catalyzes pyruvate and d-glyceraldehyde-3-phosphate (d-GAP) form 1-deoxy-d-xylulose-5-phosphate, which is then catalyzed by DXR to generate 2-*C*-methyl-d-erythritol-4-phosphate. The genes encoding *DXS* and *DXR* were first cloned and characterized in *Escherichia coli* [16,17]. Since then, *DXS* and *DXR* have been cloned in several plants, including *Arabidopsis thaliana* [18], *Zea mays* [19], and *Salvia miltiorrhiza* [20].

A multitude of studies suggest that both *DXS* and *DXR* regulate the flux through the MEP pathway and influence the accumulation of downstream products. An overexpression of *DXS* and *DXR* resulted in the greatest increase of diterpene yield found in transgenic bacteria [21]. Transgenic hairy root cultures harboring *Catharanthus roseus* *DXS* or *DXR* possessed an increased accumulation of terpenoid indole alkaloid [22,23]. Both *DXS* and *DXR* are potential regulatory sites for manipulating biosynthetic pathways to produce increased amounts of terpenoids for medicines.

Aiming at the terpenoids biosynthesis related genes in *T. wilfordii*, the *TwDXS1*, *TwDXS2*, and *TwDXR* genes were cloned and characterized in this study. In addition, the homology and evolution of these genes were analyzed, and lastly, their tissue-specific and a methyl jasmonate (MeJA)-induced expression analysis were investigated.

## 2. Results

### 2.1. Cloning and Sequence Analysis of TwDXS1, TwDXS2, and TwDXR

**Figure 1:**
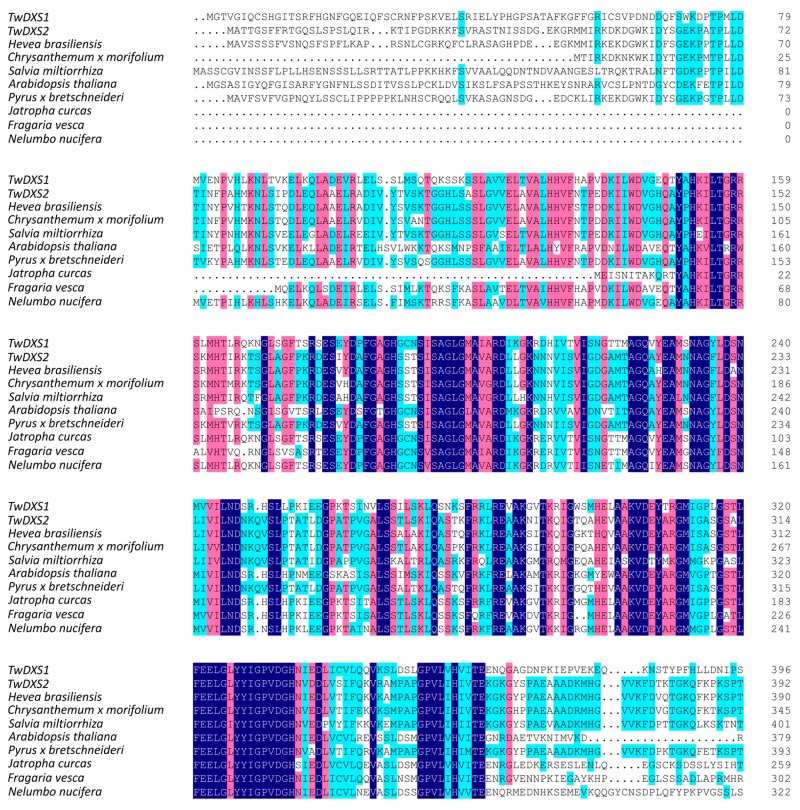

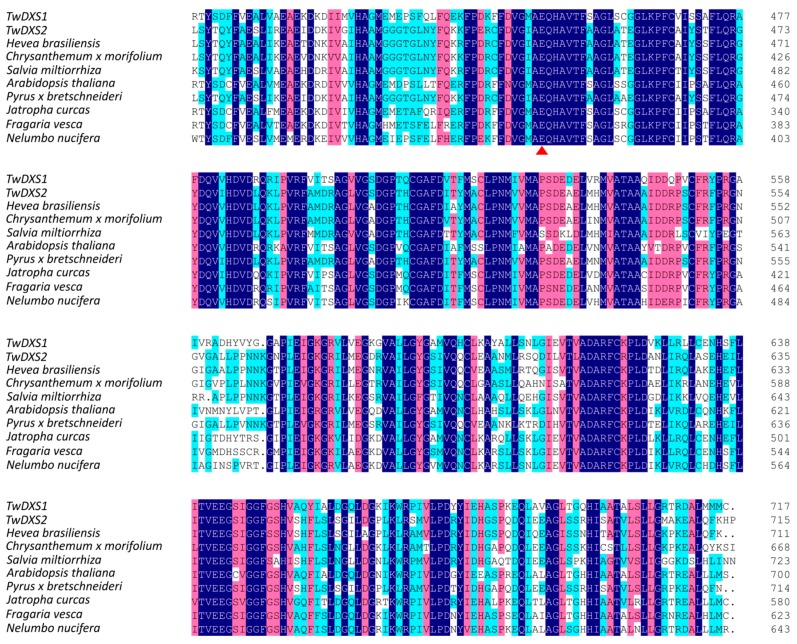
Multiple alignments of TwDXS1 and TwDXS2 with other plant DXSs. A multiple alignment of *T. wilfordii* DXS with the DXS of other plants was analyzed on DNAMAN 8.0 and based on the following protein-sequence data: *Hevea brasiliensis* (BAF98289.1), *Chrysanthemum x morifolium* (BAE79547.1), *Salvia miltiorrhiza* (ACQ66107.1), *Arabidopsis thaliana* (NP_196699.1), *Pyrus x bretschneideri* (XP_009364235.1), *Jatropha curcas* (XP_012076628.1), *Fragaria vesca* (XP_011459218.1), *Nelumbo nucifera* (XP_010254310.1). Dark blue: identity = 100%; red: 75% ≤ identity < 100%; light blue: 50% ≤ identity ≤ 75%. The functional conserved site Glu was marked with a red triangle.

**Figure 2:**
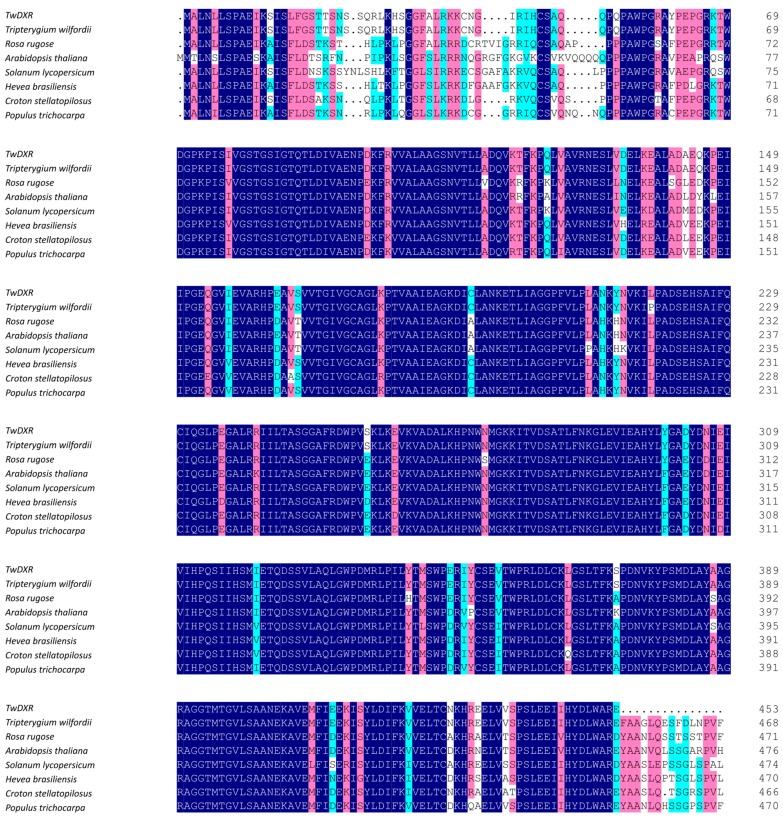
Multiple alignments of TwDXR with other plant DXRs. A multiple alignment of *T. wilfordii* DXR with the DXR of other plants was analyzed on DNAMAN 8.0 and based on the following protein-sequence data: *Tripterygium wilfordii* (AHW46302.1), *Rosa rugosa* (AEZ53171.1), *Arabidopsis thaliana* (AED97657.1), *Solanum lycopersicum* (NP_001234553.1), *Hevea brasiliensis* (ABD92702.1), *Croton stellatopilosus* (ABO38177.1), and *Populus trichocarpa* (XP_002318048.2). Dark blue boxes stand for identical residues; pink boxes stand for homologous residues. Dark blue: identity = 100%; red: 75% ≤ identity < 100%; light blue: 50% ≤ identity ≤ 75%.

The full-length cDNAs of *TwDXS1*, *TwDXS2*, and *TwDXR* were obtained and analyzed. The ORF size, number of amino acid sequence, molecular weight (*M*_W_) and isoelectric point (pI) of each deduced protein are shown in Table 1. On the amino acid level, TwDXS1 had 82%, 81%, and 80% identity to the DXS from *Jatropha curcas*, *Fragaria vesca*, and *Nelumbo nucifera*, respectively, whereas TwDXS2 was respectively 88%, 87%, and 86% identical to DXS from *Pyrus x bretschneideri*, *Hevea brasiliensis* and *Chrysanthemum x morifolium* (Figure 1). The deduced amino acid sequence of *TwDXR* exhibited a high degree of homology with the DXR sequences from other plant species, e.g., *Tripterygium wilfordii * (AHW46302.1, 99% identity), *Populus trichocarpa* (XP_002318048.2, 89% identity), *Croton stellatopilosus* (ABO38177.1, 88% identity), and *Hevea brasiliensis* (ABD92702.1, 87% identity) (Figure 2).

**Table 1:** Sequence information for *DXS* and *DXR*.

Gene Name	Full Length (bp)	ORF (bp)	Amino Acid Sequence (aa)	*M*_W_ (kDa)	pI
*Tw DXS1*	2556	2154	717	78.7	6.04
*Tw DXS2*	2412	2148	715	77.0	7.10
*Tw DXR*	1816	1410	469	51.1	5.88

### 2.2. Bioinformatics Analysis

The Interpro results showed that the molecular function of both TwDXS1 and TwDXS2 were 1-deoxy-d-xylulose-5-phosphate synthase activity. The structure of the DXS monomer contained three domains including thiamine diphosphate-binding fold, pyrimidine binding domain (transketolase-like) and transketolase C-terminal domain. It has been reported that the catalysis of DXS required the coenzyme thiamine pyrophosphate (TPP) and thiamine diphosphate-binding domain. This domain contained the corresponding binding site, like the conserved residues 228–259 and 454–544, which were crucial for catalysis in *Deinococcus radiodurans* [24]. An invariant glutamate (Glu) residue in positions 454–544 is believed to be required for transketolase and transketolase-like activity [25,26]. In multiple alignments of the DXSs, the functional conserved site Glu were found in all DXS sequences (Figure 1).

**Figure 3:**
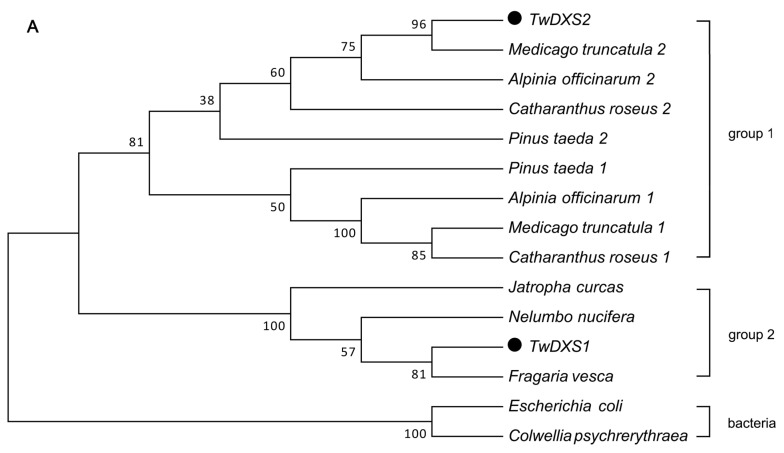

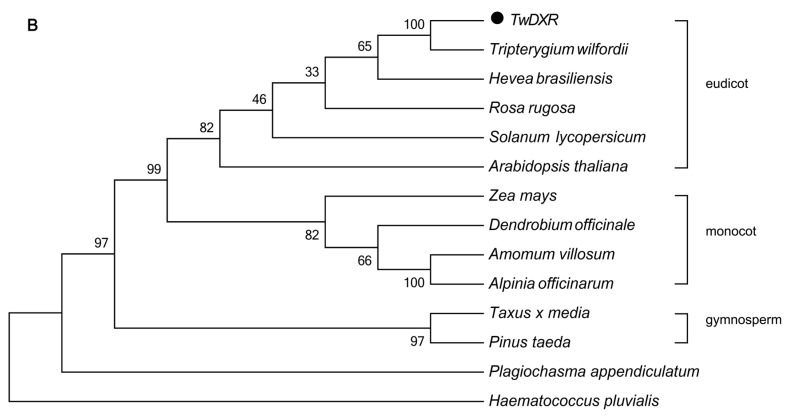
Phylogenetic analysis of the amino acid sequences of *DXSs* (**A**) and *DXRs* (**B**). The Neighbor-Joining phylogenetic trees were constructed using the bootstrap method on MEGA 5.1 and the number of Bootstrap replications was 1000. The protein sequences used in these trees are as follows: (**A**) *DXS*: *Alpinia officinarum* 1 (AEK69518.1), *Alpinia officinarum* 2 (AEK69519.1), *Medicago truncatula* 1 (CAD22530.1), *Medicago truncatula* 2 (CAD22531.1), *Pinus taeda* 1 (ACJ67021.1), *Pinus taeda* 2 (ACJ67020.1), *Catharanthus roseus* 1 (ABI35993.1), *Catharanthus roseus* 2 (AGL40532.1), *Jatropha curcas* (XP_012076628.1), *Fragaria vesca* subsp. *Vesca* (XP_011459218.1), *Nelumbo nucifera* (XP_010254310.1), *Escherichia coli* (AIZ85961.1), *Colwellia psychrerythraea* (KGJ90592.1); (**B**) *DXR*: *Tripterygium wilfordii* (AHW46302.1), *Rosa rugosa* (AEZ53171.1), *Arabidopsis thaliana* (AED97657.1), *Solanum lycopersicum* (NP_001234553.1), *Amomum villosum* (ACS26204.1), *Zea mays* (ACG33012.1), *Hevea brasiliensis* (ABD92702.1), *Haematococcus pluvialis* (AEY80027.1), *Taxus x media* (AAU87836.1), *Pinus taeda* (ACJ67022.1), *Plagiochasma appendiculatum* (AFM78686.1), *Dendrobium officinale* (AGT29340.1), *Alpinia officinarum* (AEK69520.1).

The evolutionary position of *TwDXS1*, *TwDXS2* and *TwDXR* were shown in a phylogenetic tree of the DXSs (Figure 3A) and DXRs (Figure 3B). The cluster relation of DXSs phylogenetic tree consistent with the species taxonomy. *TwDXS1* and *TwDXS2* belonged to plant clade and developed group 1 and group 2 sub-branches. The *TwDXS2* was more identical to *DXS2* from *Medicago truncatula*, *Alpinia officinarum*, *Catharanthus roseus*, and *Pinus taeda*; while *TwDXS1* gathered into a new clade. The DXRs derived from an ancestral gene and evolved into five groups including the green algae, liverworts, pinales, monocots, and eudicots groups, which were suggestive of an evolutionary relationship from lower plants to higher plants. In the eudicots group, *TwDXR* was nearly identical to *T. wilfordii* DXR. The bioinformatics analysis strongly suggested that *TwDXR* was a plant DXR protein with 1-deoxy-d-xylulose-5-phosphate reductoisomerase activity.

### 2.3. Color Complementation and Functional Analysis

**Figure 4:**
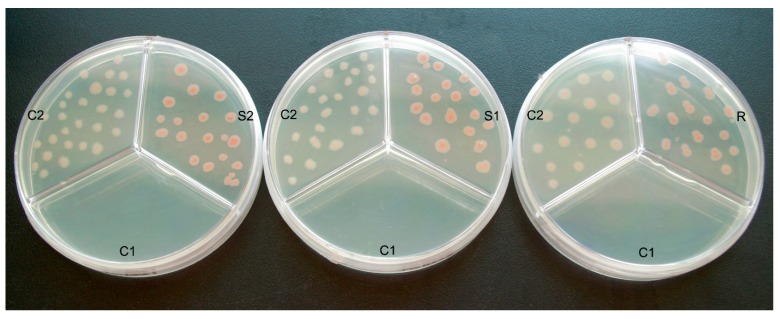
Functional identification of *TwDXS1*, *TwDXS2*, and *TwDXR* in *E. coli* Trans1-Blue cells. The pTrc-*TwDXS1* (S1), pTrc-*TwDXS2* (S2) and pTrc-*TwDXR* (R) were respectively co-transformed into the *E. coli Trans*1-Blue cells with pAC-LYC. Single transformation of pAC-LYC was used as C1. The *E. coli* cells with pTrc and pAC-LYC were treated as C2. All of them were co-cultured in the LB medium with 150 mg·L^−1^ Ap and 50 mg·L^−1^ Chl.

Since Francis X. Cunningham constructed plasmid pAC-LYC [27], it has been used for functional analyses of genes in terpenoid biosynthetic pathway. Via treating the lycopene accumulating strains of *E. coli* as heterologous hosts, researchers have identified several cDNAs encoding enzymes of isoprenoid biosynthesis, like the glyceraldehydes-3-phosphate dehydrogenase gene from *Arabidosis thaliana* and Z*ea mays* [28,29], the *DXS* from Marigold and *Adonis aestivalis* [30,31], the *DXR* from *Salvia miltiorrhiza* [32], the isopentenyl-diphosphate-isomerase from *H. pluvialis* and *Zea mays* [29,33]. To identify the functions of *TwDXS1*, *TwDXS2*, and *TwDXR*, the color complementation method was performed. Compared to the control 2 (C2) cells with the empty vectors pTrc and pAC-LYC, *E. coli* *Trans*1-Blue cell containing pTrc-*TwDXS1* and pAC-LYC formed a pinker colour of colonies. The control 1 (C1), *E. coli* cells harboring single vector pAC-LYC, were unable to grow on Luria-Bertani (LB) medium with ampicillin (Ap) and chloramphenicol (Chl) due to lack of the Ap resistance. The color complementation results of *TwDXS2* and *TwDXR* were similar to the *TwDXS1* (Figure 4).

The light and dark color of colonies revealed the yield of lycopene directly, and obviously, the more lycopene the colonies produced, the pinker the color would be. Hence, it was observed that *E. coli* cells containing pTrc-*TwDXS1* and pAC-LYC accumulated higher concentrations of lycopene than the C2. Heterologous expression of the *TwDXS1* gene accelerated the production of lycopene, thus the cells formed hot pink colonies. Similar analyses were used to identify the functions of *TwDXS2* and *TwDXR*. The results showed that heterologous expression of *TwDXS2* and *TwDXR* promoted lycopene synthesis, as evidenced by hot pink colonies, and even some red colonies (Figure 4). The color complementation assay confirmed that *TwDXS1*, *TwDXS2* and *TwDXR* encoded functional proteins and played a crucial role in promoting lycopene pathway flux.

### 2.4. Tissue-Specific Expression Analysis

**Figure 5:**
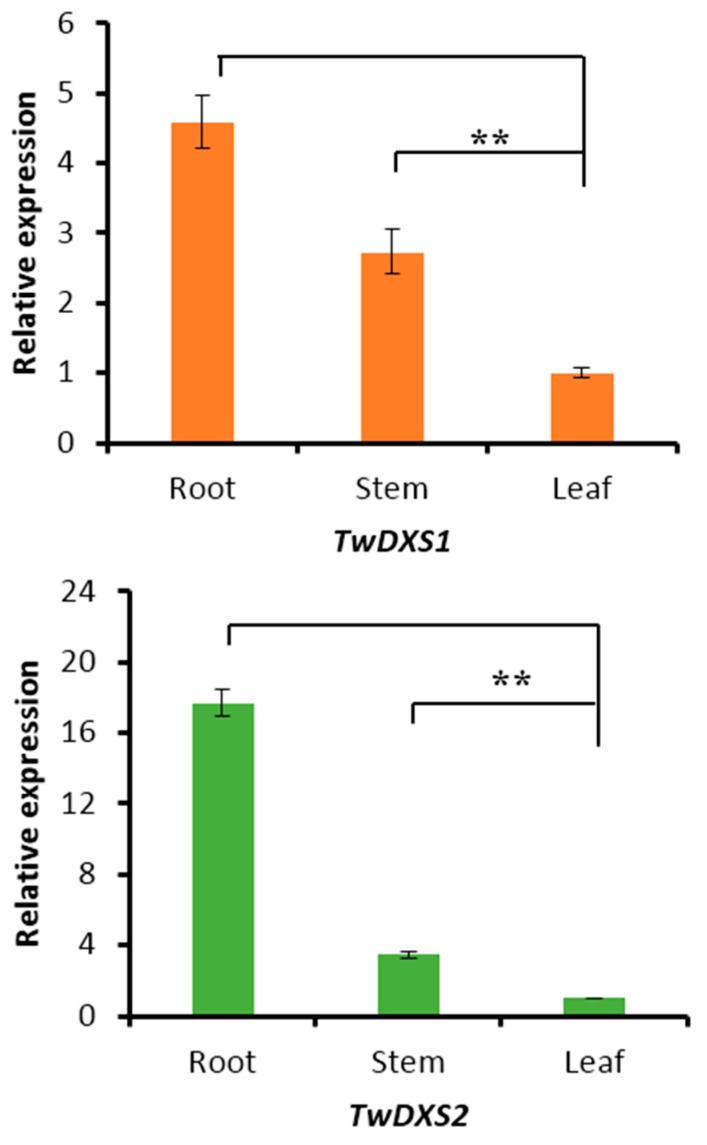
Expression patterns of *TwDXS1* and *TwDXS2* in different tissues of *T. wilfordii* aseptic seedling. The data represented the average of four independent experiments each carried out in triplicate; the error bars showed standard deviations. The asterisks indicated that the difference is significant compared with leaf (** *p* < 0.05).

The transcript levels of *TwDXS1*, *TwDXS2*, and *TwDXR* were analyzed using quantitative real-time polymerase chain reaction (qRT-PCR). The tissue-specificity results in *T. wilfordii* aseptic seedlings showed that both *TwDXS1* and *TwDXS2* were expressed extensively in all of the examined tissues, including photosynthetic and non-photosynthetic tissues (Figure 5). The roots were found containing the highest levels of *TwDXS1* and *TwDXS2* gene expression. For *TwDXS2*, the expression level in the roots was almost 18-fold higher than that in the leaves, and 5-fold higher than that in the stems. The expression of *TwDXS1* revealed relatively moderate changes across different tissues. The roots were more than four times as likely to express *TwDXS1* as the leaves, and twice as likely to express *TwDXS1* as the stems. All of the above results suggested that *TwDXS1* and *TwDXS2* are constitutively expressed genes, similar to what has been reported for *Arabidopsis* [34], *Amomum villosum* [35] and *Aquilaria sinensis* [36]. However, the transcript levels of *TwDXR* were somewhat low and could not be detected using the qRT-PCR in all of the tested tissues.

### 2.5. Transcript Level of DXS/DXR in Suspension Cells and Metabolite Analysis

**Figure 6:**
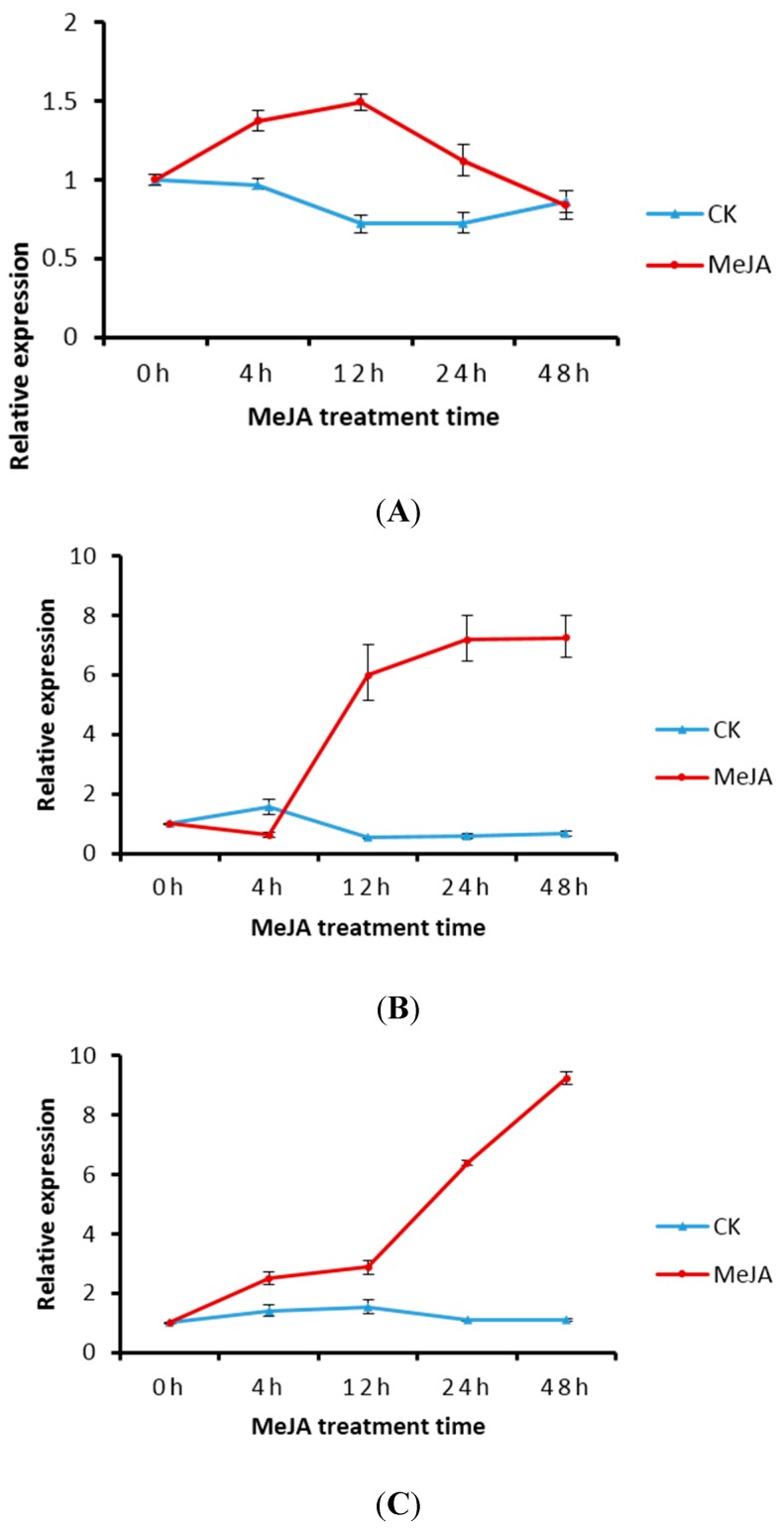
Expression of *TwDXS1* (**A**); *TwDXS2* (**B**) and *TwDXR* (**C**) in *T. wilfordii* suspension cells after MeJA induction. The data represented the average of four independent experiments each carried out in triplicate; the error bars showed standard deviations. MeJA: methyl jasmonate treated group; CK: untreated control group.

The expressions of *TwDXS1*, *TwDXS2* and *TwDXR* in *T. wilfordii* suspension cells were increased after treatment with MeJA. The expression of *TwDXS1* was enhanced at 4 and 12 h via MeJA treatment, and then decreased (Figure 6A), which was similar to a previous report on Indian ginseng [37]. The expression of *TwDXS2* declined during the first 4 h and was then continuously upregulated to approximately 7.24-fold higher than the control levels at 48 h (Figure 6B). The *TwDXR* expression increased sustainably with a rapid rise after 12 h. The highest level of *TwDXR* expression was observed after 48 h of 50 μmmol·L^−1^ MeJA treatments, which resulted in an approximately 9.22-fold higher level of expression (Figure 6C).

**Figure 7:**
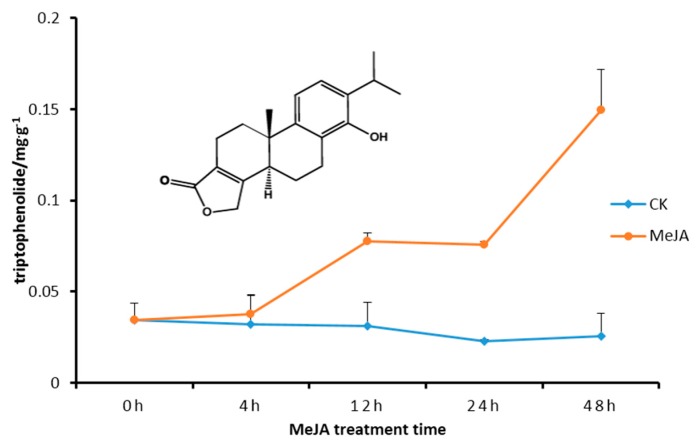
Triptophenolide contents in *T.wilfordii* suspension cells after treatment with MeJA. The data represented the average of four independent experiments each carried out in triplicate; the error bars showed standard deviations.

As shown in Figure 6, the *TwDXS1*, *TwDXS2*, and *TwDXR* expression were increased in the MeJA treated cultures. A similar rising trend about the triptophenolide content was found with the expression level variation. The metabolite analysis revealed that MeJA treatment improved the content of triptophenolide at 12–48 h, reaching levels nearly six-fold higher at 48 h (Figure 7). The increasing triptophenolide level variation correlated with the change of *TwDXS2* and *TwDXR* mRNA levels. The results indicated that the upregulating expression of *TwDXS2* and *TwDXR* enhanced the synthesis of triptophenolide in suspension cells.

## 3. Discussion

The color complementation methods have been used for identifying the functions of DXSs, DXRs, and HMGRs enzymes, like in *Salvia miltiorrhiza*, *Amomum villosum*, and *Camptotheca acuminata* [32,35,38]. Prior to the official color experiments, the *E. coli* strains Trans 5α, Top 10, Trans1-Blue, DH 10B, BL21 were tested for lycopene accumulation. The BL21 strains produced virtually no lycopenes, just as Cunningham [28] reported. The suggested reason for this is that BL21 strains easily form colonies with sectors that are deficient in lycopene accumulation. *E. coli* Trans1-Blue cells, which assist in the accumulation of lycopenes, were chosen for the functional characterization. Due to a lack of carotenogenic gene clusters, *E. coli* bacteria cannot generally produce lycopene. The addition of pAC-LYC helped increasing the yield of lycopene, consequently the colonies displayed pink color [39]. The darker color helps to demonstrate an increasing metabolic flux to the synthesis of isoprene compounds.

The DXSs are the critical enzyme catalyzing the first committed step of the MEP pathway, the genes coding for the rate-limiting enzymes may play a crucial role in synthesizing isopentenyl pyrophosphate and downstream various final terpene products. Many published reports of experimental data confirmed that the transcript levels were correlated with terpene accumulation in *Ginkgo biloba* [40], and tomato [41] *etc*. In plants, the *DXS* multigene families comprise three independent classes by functional division. One is specifically involved in the synthesis of essential terpenoids like photosynthetic pigments [34]; one was to synthesize the secondary metabolites of specific terpenoids; the last one participates in the synthesis of indispensable isoprenoids such as phytohormones that are required at low levels. A previous study showed that *cla1* mutants in *Arabidopsis thaliana* with the albino phenotype could not be rescued by *DXS2* and *DXS3* [42]. In addition, it has been shown that multicopy *DXSs* are conserved in plants prior to the monocot-dicot divergence. *DXS2* expression was involved in feedback mechanisms, and its down-regulation led to an increase in sesquiterpenes [43]. DXSs, particularly the most divergent of the three classes, present negative feedback to the inhibition of lycopene pathway flow [44]. In addition to DXS, studies in plants suggested that the second enzyme of the MEP pathway, DXR, also has a rate-limiting role in IPP and DMAPP synthesis, and isoprenoid biosynthesis. In maize, DXR protein accumulation is tightly correlated with the abundance of carotenoid [19]. The transplastomic tobacco plant containing *DXR* genes from *Synechosystis* sp. strain PCC6803 showed increase in the content of isoprenoids such as *β*-carotene, lutein, and solanesol [45].

As the first two committed enzymes in the pathway, DXS and DXR are directly involved in the formation of MEP, which contains the characteristic branched C5 skeleton and is so far not found to be the precursor of other metabolic pathways [46,47]. Thus MEP can be the first significant intermediate, and DXS/DXR play a crucial role in flux regulation of the MEP pathway. Metabolic regulation research suggested that feedback on the activity of the first enzyme DXS from the last metabolites, IPP and DMAPP, potentially contributes an important mechanism of the MEP pathway regulation. The IPP and DMAPP competed with thiamine pyrophosphate for binding with the DXS [48,49]. Another feedforward regulation at DXS showed that d-GAP accelerated the rate of pyruvate decarboxylation by 600-fold [50]. Additionally, transgenic alterations of DXS activity in *Arabidopsis thaliana* showed that this enzyme has a very high flux control coefficient for the MEP pathway [51].

Triptophenolide has obvious bioactivity, and also can be used as starting material to synthesize triptolide and triptonide. However, triptophenolide level was 0–67.7 µg·g^−1^ in *T. wilfordii* roots, which is far from enough for clinical use [52]. Plant metabolic engineering provides alternative strategies to improve the productivity of secondary metabolites. The use of elicitors such as MeJA, yeast elicitor, and salicylic acid is one of the attractive approaches and suitable for cell lines and hairy roots. The MeJA used in this study improved the triptophenolide in suspension cells to 149.52 µg·g^−1^, a 120% increase compared with the roots. *Salvia miltiorrhiza* hairy roots harboring *DXS* produced higher level of tanshinone (0.867 to 3.031 mg·g^−1^) compared with the control line (0.613 mg·g^−1^) [53]. Co-expression of a *DXS* from tomato and a geranylgeranyl diphosphate synthase from tobacco in *Nicotiana benthamiana* led to a 3.5-fold increase in the amount of cembratrien produced, with maximum yields reaching 2500 ng·cm^−^^2^ [54]. Hence, it will be an interesting and effective strategy to improve triptophenolid content by genetic engineering. However, the transgenic regeneration system of *T. wilfordii* remains unsolved, and further research is needed. The cloning and identification of *TwDXSs* and *TwDXR* will assist us in enhancing the production of terpenoids in *T. wilfordii*.

## 4. Experimental Sections

### 4.1. Plant Materials

Using methods previously described [55,56], the white and soft calluses came from explants and were cultured in Murashige and Skoog (MS) agar medium (Sigma, St. Louis, MO, USA) with 0.5 mg·L^−1^ 2,4-Dichlorophenoxyacetic acid (2,4-D) (Sigma), 0.5 mg·L^−1^ Indole-3-butytric acid (IBA) (Sigma), and 0.1 mg·L^−1^ Kinetin (KT) (Sigma) at 25 °C in the dark. Then the calluses were placed in MS agar medium containing 0.2 mg·L^−1^ indole-3-acetic acid (IAA), 0.5 mg·L^−1^·KT, and 1.5 mg·L^−1^ 6-benzylaminopurine (6-BA) (Sigma) and incubated in a photoperiod of 16 h light and 8 h dark at 25 °C to develop aseptic seedlings. Aseptic seedlings used in this study were cultured about six month. The calluses were also used to initiate *T. wilfordii* suspension cells grown in MS medium supplemented with 0.5 mg·L^−1^ 2,4-D, 0.5 mg·L^−1^ IBA, 0.1 mg·L^−1^·KT and 30 g·L^−1^ sucrose, in the dark at 25 °C with rotary shaking at 120 rpm, and transferred frequently at 20 day intervals.

### 4.2. RNA Isolation and cDNA Cloning

Ten-day-old *T. wilfordii* suspension cells were treated with MeJA in MS medium at a final concentration of 50 μmol·L^−1^. Untreated suspension cells were used as a control. The suspension cells were harvested at 0, 12, 24, and 48 h. Each experiment was independently replicated 3 times. All of the samples were immediately frozen in liquid nitrogen and stored at −80 °C prior to RNA isolation. The total RNA was extracted via the modified cetyltrimethylammonium bromide (CTAB) method [56] and purified using RNase-free DNaseI according to the manufacturer’s instructions (RNA Purification Kit, TianGen Bio., Beijing, China). The specific cDNA isolations were obtained through the PrimeScript 1st Strand cDNA Synthesis Kit (Takara Bio Inc., Dalian, China), the SMART™ RACE cDNA Amplification Kit (Clontech, Dalian, China) and PrimeSTAR GXL DNA Polymerase (Takara Bio Inc) according to the manufacturer’s instructions. Depending on the initial *TwDXS1*, *TwDXS2* and *TwDXR* sequences in transcriptome data, the primers were designed (Table 2), subsequently 3′-RACE and LC-PCR were performed for cloning the full length of *TwDXS1*, *TwDXS2* and *TwDXR*. The PCR products were purified and ligated into pMD 19-T (Takara Bio Inc.). Subsequently, the total products were transformed into *E. coli* DH5α competent cells (TransGen Bio., Beijing, China) and the plasmid DNA was isolated and sequenced (Majorbio, Beijing, China).

**Table 2:** Primers used in this study.

Usage	Primer Name	Primer Sequences 5′→3′
RACE-PCR	DXR-3′	GTGTATTCAAGGATTGCCAGAGGGTG
LC-PCR	DXS1-F	AGACTCGAATTATGCTCCGTTT
	DXS1-R	GCAGCATTATACATTTTTCTACGG
	DXS2-F	AAGGGAGGATGATGATACGCA
	DXS2-R	CTCATAGCATGTATGGTGCTAGAGC
	DXR-F	CCCGAACCCAAACCACAC
	DXR-R	AGGAGTAGATTAACCGGGATGAT
ORF-PCR	ORFDXS1-F	GGAAGATCTATGGGTACTGTTGGTATTCAGTGCTC
	ORFDXS1-R	TTGCGGCCGCCTAGCACATCATC
	ORFDXS2-F	GGAAGATCTATGGGTACTGTTGGTATTCAGTGCTC
	ORFDXS2-R	CGGGGTACCATGGCGACTACTGGGTCTTTC
	ORFDXR-F	CGCGGATCCTTAGGGATGCTTAAACT
	ORFDXR-R	TTGCGGCCGCTCATGCAAAGACAGGATTTA
qRT-PCR	RTDXS1-F	CTCCTTAGGCTGCTATGTGA
	RTDXS1-R	GCTGTTCCTTGGGTGATGC
	RTDXS2-F	GGGCATCAGGCATACCCACACAAG
	RTDXS2-R	TGGCTCCATCTCCAATCAC
	RTDXR-F	TCAAGGATTGCCAGAGGG
	RTDXR-R	ATGAATGATAGACTGCGGATG
	Actin-F	AGGAACCACCGATCCAGACA
	Actin-R	GGTGCCCTGAGGTCCTGTT

The underlined bases represent the restriction sites used for cloning.

### 4.3. Data Analysis

The sequences of the genes were first confirmed online (Available online: http://www.ncbi.nlm.nih.gov/), and their nucleotide sequences, deduced amino acid sequences and open reading frames (ORFs) were analyzed. The theoretical pIs and *M*_W_s of the deduced proteins were computed with the Compute pI/*M*_W_ Tool (Available online: http://web.expasy.org/compute_pi/). Interpro (Available online: http:// www.ebi.ac.uk/interpro/scan.html) was used to analyze protein sequences. A multiple alignment analysis of the amino acid sequences was carried out using DNAMAN8.0 software (Lynnon Biosoft, Quebec, QC, Canada). Phylogenetic trees of TwDXS and TwDXR and several other plants were constructed using MEGA5.1 software (Arizona State University, Tempe, AZ, USA). Predictions of the functions of the deduced proteins were performed using conserved domains (Available online: http://www.ncbi.nlm.nih.gov/Structure/cdd/wrpsb.cgi).

### 4.4. Construction of Expression Vectors

The pTrc-*AtIpI* and pAC-LYC vectors were kindly provided by Francis X. Cunningham Jr. (Department of Cell Biology and Molecular Genetics, University of Maryland, College Park, MD, USA)*.* The pTrc-*AtIPI* vector was the combination of isopentenyl diphosphate isomerase (*IPI*) gene from *Arabidopsis thaliana* (*AtIPI*), pTrcHisB and pBlueScript SK-. More specifically, the pTrcHisB vector was digested by restriction enzyme *Kpn*I and *EcoR*I, and then the coding sequence of *AtIPI* inserted. Subsequently, the 6× His tag was removed, as well as the fragment located between *EcoR*V and *EcoR*I. The fragments with an *AtIPI* gene and Trc promoter were finally digested using *EcoR*V and *EcoR*I and inserted into the pBlueScript SK-plasmid [57,58]. The pTrc-*AtIPI* vector, as an expression vector, retains an Ap resistance gene. On the other hand, the pAC-LYC plasmid contains all of the functional genes involved in lycopene biosynthesis, including the geranylgeranyl pyrophosphate synthase (*crtE*), phytoene synthase (*crtB*), and phytoene desaturase (*crtI*) genes [27]. *E. coli* cells containing pAC-LYC vectors can survive in LB medium with Chl and produce small amounts of lycopene, resulting in light pink colonies.

In order to respectively insert the coding region cDNA fragments of *TwDXS1*, *TwDXS2* and *TwDXR* to pTrc-*AtIPI* vectors, the double digestion and ligation reactions were performed. More specifically, the primers were located in the ends of ORF and added restriction sites (Table 2) to amplify the coding region of *TwDXS1*, *TwDXS2* and *TwDXR*. The amplification reactions contained 10 μL of 5× PrimerSTAR GXL buffer, 4 μL of dNTP (2.5 mM), 1 μL of each primer (10×), 1 μL of PrimerSTAR GXL DNA Polymerase and 1 μL of DNA template in a 50 μL total volume. The PCR conditions were as follows: 98 °C for 3 min; 35 cycles at 98 °C for 10 s, 60 °C for 15 s, 68 °C for 2 min and 20 s (for DXS)or 1 min and 30 s (for DXR); 68 °C for 5 min; the sample were then held at 4 °C. Next, the PCR products were purified using a Gel Extraction Kit (TianGenBio.). Both pTrc-*AtIPI* vectors and ORF-PCR fragments were digested by identical pairs of restriction endonucleases (*Kpn*I, *BamH*I for DXS1; *Bgl*II, *Not*I for DXS2; *Bgl*II, *Not*I for DXR) and ligated with Quick T4 DNA ligase (Takara Bio. Inc.). The final recombination expression vectors, pTrc-*TwDXS1*, pTrc-*TwDXS2* and pTrc-*TwDXR* were sequenced and extracted (GV-Plasmid DNA Mini Extraction Kit, TianGenBio.). As a negative control, the pTrc-*AtIPI* vector was digested with *Pst*I to remove *AtIPI* [35] and connected into pTrc vector.

### 4.5. Color Complementation

To identify the function of *TwDXS1*, the pTrc-*TwDXS1* and pAC-LYC vectors were co-transformed into *Trans*1-Blue chemically competent *E. coli* cells (TransGen Biotech, Beijing, China). Meanwhile, pTrc and pAC-LYC were also co-transformed into *Trans*1-Blue *E. coli* as control 1 and single pAC-LYC was transformed as control 2. To ensure identical culture conditions, the three kind of *E. coli* cells were coated in one culture dish with LB medium containing Ap (150 mg·L^−1^) and Chl (50 mg·L^−1^). All of the LB media were positioned upside down at 37 °C for 16 h and then kept at room temperature for 2–5 days for maximal color development [28,39]. The functional identification of pTrc-*TwDXS2* and pTrc-*TwDXR* were performed in the same manner as pTrc-*TwDXS1*. The colors of all the transformants were observed to characterize the function of each target gene.

### 4.6. Tissue-Specific Expression Analysis

For the tissue-specific expression analysis, qRT-PCRs were performed using an Applied Biosystems 7300 (Applied Biosystems, Foster City, CA, USA) machine. The total RNA was isolated from the roots, stems and leaves of four *T. wilfordii* aseptic seedlings. All of the RNAs were converted to first-strand cDNAs as templates for qRT-PCR. The house-keeping gene β-actin was used as internal control in each reaction. The relevant primers for *TwDXS1*, *TwDXS2* and *TwDXR* were designed in the coding sequences as shown in Table 2. The amplifications were carried out in 20 μL of reaction mix containing 1 μL of genomic DNA, 10 μL of 2× qPCR Master Mix, 0.4 μL of 50× ROX reference Dye High (KaPa Biosystems, Wilmington, DE, USA), and 0.4 μL of primers (10 μm). The qRT-PCR conditions consisted of initial denaturation for 5 min at 95 °C, followed by denaturation 3 s at 95 °C, annealing and elongation for 30 s at 60 °C. These conditions were repeated for 40 cycles. The relative gene expression level was calculated using the 2^−∆∆*C*t^ method [59].

### 4.7. Elicitation of Suspension Cells with MeJA

The concentration of MeJA in the culture was fixed at 50 μmol·L^−1^. *T. wilfordii* suspension cells were treated with MeJA and collected at 0, 4, 12, 24 and 48 h after the application of MeJA for analysis of the RNA level and the total triptophenolide content. The qRT-PCR method used in the tissue-specific expression analysis was conducted to detect the expression level of *TwDXS1*, *TwDXS2* and *TwDXR* in suspension cells induced by MeJA*.* Meanwhile, all the samples were freeze-dried for 8 h and extraction was performed according to a previously reported method [55]. The extracts were analyzed by an Agilent 1260 HPLC system (USA) on an Agilent Eclipse XDB-C18 analytical column (5 μm, 4.6 mm × 250 mm; Agilent, Santa Clara, CA, USA) at 35 °C. The mobile phase used in this study was a mix of solvent A (water) and solvent B (acetonitrile). The program was 38% solvent B for 12 min. The flow rate of the solvent was kept constant at 0.8 mL·min^−1^. The injection volume was 10 μL and the samples were detected at wavelengths of 210 nm.

## 5. Conclusions

In conclusion, we have successfully isolated the *DXS1*, *DXS2* and *DXR* genes in *T. wilfordii* and identified their functions using color complementation experiments. In addition, expression analyses were performed by qRT-PCR analysis. The metabolite analyses indicated that *TwDXS* and *TwDXR* played an important role in terpenoid biosynthesis in *T. wilfordii*. These results provided the basis for further work including the regulation of these genes, characterization of downstream genes, and biosynthesis of terpenoid natural products and finally created new approaches for their production.
